# Focused Ultrasound-Enhanced Liquid Biopsy: A Promising Diagnostic Tool for Brain Tumor Patients

**DOI:** 10.3390/cancers16081576

**Published:** 2024-04-19

**Authors:** Akke Bakker, Anna E. Ixkes, Hema Venugopal, Mario G. Ries, Nathalie S. M. Lak, Filip Y. F. L. de Vos, Dannis G. van Vuurden, Tom J. Snijders

**Affiliations:** 1 Princess Máxima Center for Pediatric Oncology, Heidelberglaan 25, 3584 CS Utrecht, The Netherlands; 2 Department of Neurology & Neurosurgery, UMC Utrecht Brain Center, UMC Utrecht, Heidelberglaan 100, 3584 CX Utrecht, The Netherlands; 3 Biomedical Sciences, Utrecht University, Heidelberglaan 8, 3584 CS Utrecht, The Netherlands; 4 Imaging Division, UMC Utrecht, Heidelberglaan 100, 3584 CX Utrecht, The Netherlands; 5 Department of Medical Oncology, UMC Utrecht, Heidelberglaan 100, 3584 CX Utrecht, The Netherlands

**Keywords:** focused ultrasound, microbubbles, blood-brain barrier opening, liquid biopsy, biomarkers, brain tumors

## Abstract

**Simple Summary:**

Diagnosing brain tumors using minimally invasive methods, such as a liquid biopsy, is challenging due to the blood–brain barrier (BBB). The BBB blocks tumor biomarkers from entering the bloodstream. However, a technique called focused ultrasound with microbubbles (FUS-BBBO) can temporarily open the BBB, thereby potentially increasing the tumor biomarkers in the bloodstream. This systematic review collected data on FUS-BBBO-enhanced liquid biopsy for primary brain tumors. The review included five animal studies and two human studies. Animal studies have shown that biomarker levels were higher in groups subjected to FUS-BBBO compared to control groups. Clinical studies involving 14 patients also showed increased biomarker levels after FUS-BBBO treatment. It is worth noting that using stable cavitation during FUS-BBBO appeared promising for liquid biopsy. Overall, this technique has the potential to improve brain tumor diagnosis and disease monitoring. However, further investigation is necessary to ensure its safe and effective use in the clinical setting.

**Abstract:**

The performance of minimally invasive molecular diagnostic tools in brain tumors, such as liquid biopsy, has so far been limited by the blood–brain barrier (BBB). The BBB hinders the release of brain tumor biomarkers into the bloodstream. The use of focused ultrasound in conjunction with microbubbles has been shown to temporarily open the BBB (FUS-BBBO). This may enhance blood-based tumor biomarker levels. This systematic review provides an overview of the data regarding FUS-BBBO-enhanced liquid biopsy for primary brain tumors. A systematic search was conducted in PubMed and Embase databases with key terms “brain tumors”, “liquid biopsy”, “FUS” and their synonyms, in accordance with PRISMA statement guidelines. Five preclinical and two clinical studies were included. Preclinical studies utilized mouse, rat and porcine glioma models. Biomarker levels were found to be higher in sonicated groups compared to control groups. Both stable and inertial microbubble cavitation increased biomarker levels, whereas only inertial cavitation induced microhemorrhages. In clinical studies involving 14 patients with high-grade brain tumors, biomarker levels were increased after FUS-BBBO with stable cavitation. In conclusion, FUS-BBBO-enhanced liquid biopsy using stable cavitation shows diagnostic potential for primary brain tumors. Further research is imperative before integrating FUS-BBBO for liquid biopsy enhancement into clinical practice.

## 1. Introduction

Malignant primary brain tumors account for less than 1% of all primary malignant tumors in adults in the United States, but they are the most commonly diagnosed solid tumors in children and adolescents [1,2]. Malignant primary brain and other central nervous system (CNS) tumors are the leading cause of cancer death among males aged <40 years and females aged <20 years [2]. Glioblastoma is the most prevalent malignant primary brain tumor in adults, with a five-year survival rate of less than eight percent [3]. In children, brain tumors are the most common solid neoplasms and the leading cause of death from cancer [1,4,5]. Treating patients with brain tumors remains challenging, which can be partly attributed to impaired drug delivery to the parenchyma. The blood–brain barrier (BBB) is a selectively permeable membrane that separates the blood pool from the brain parenchyma. The entry of many small and, even more so, large drugs into the CNS and, thus, brain tumors is hindered by restricted passive diffusion through the BBB and/or active efflux transporters, which both limit bioavailability in the parenchyma [6]. As a result, many promising and innovative drug treatments effective in vitro cannot be translated into effective treatments for brain tumor patients. 

Molecular-level diagnosis of brain tumors is essential for both effective treatment and predicting long-term prognosis. Tissue analysis obtained by an intracranial surgical procedure is the standard method for obtaining this information. However, this invasive procedure is complex, costly and carries inherent risks, including bleeding, infection, brain damage, seizures and stroke [7], in up to 8% of patients [8]. Before a patient with a brain tumor undergoes a surgical biopsy, the potential risks and benefits are carefully considered. In some cases, the risks may outweigh the benefits of having an accurate diagnosis. Repeated tissue resection is not performed structurally because of this risk–benefit ratio; however, repeated histological sampling can be valuable to discriminate the true progression of tumor from radiation necrosis and other treatment effects [9], as well as providing insight into therapy resistance. In fact, diffuse gliomas often show an evolution of molecular phenotype over time [9]. Molecular phenotyping of a progressive primary or recurrent brain tumor may aid the choice of treatment. Liquid biopsy offers the ability to gather important molecular information for diagnosis, prognosis and disease monitoring with minimal invasion through either blood or CSF sampling [10]. For longitudinal assessment, a blood sample is generally preferred since it is less invasive and can be repeated numerous times [11]. 

Common analytes of liquid biopsy for cancer include circulating tumor cells (CTC), extracellular vesicles (EV), proteins, circulating tumor DNA (ctDNA) and RNA, e.g., messenger-RNA (mRNA) and microRNA (miRNA) (Table 1) [12]. These biomarkers carry pathogenic signatures, such as DNA mutations, epigenetic alterations, DNA copy number alterations and microRNA expression profiles [10]. Despite the considerable potential so far, the role of liquid biopsies in clinical care for brain tumors is still limited. The quantity of tumor-derived biomarkers present in a blood sample is, at present, insufficient for accurate diagnosis. A study comparing blood-borne ctDNA levels in different cancer types found that less than 10% of patients with gliomas had detectable ctDNA, compared to more than 75% of patients with different types of metastatic non-CNS-cancers [13]. In addition to differences in the tumor-shedding potential, this could be due to the presence of an intact BBB [14,15].

**Figure 1:**
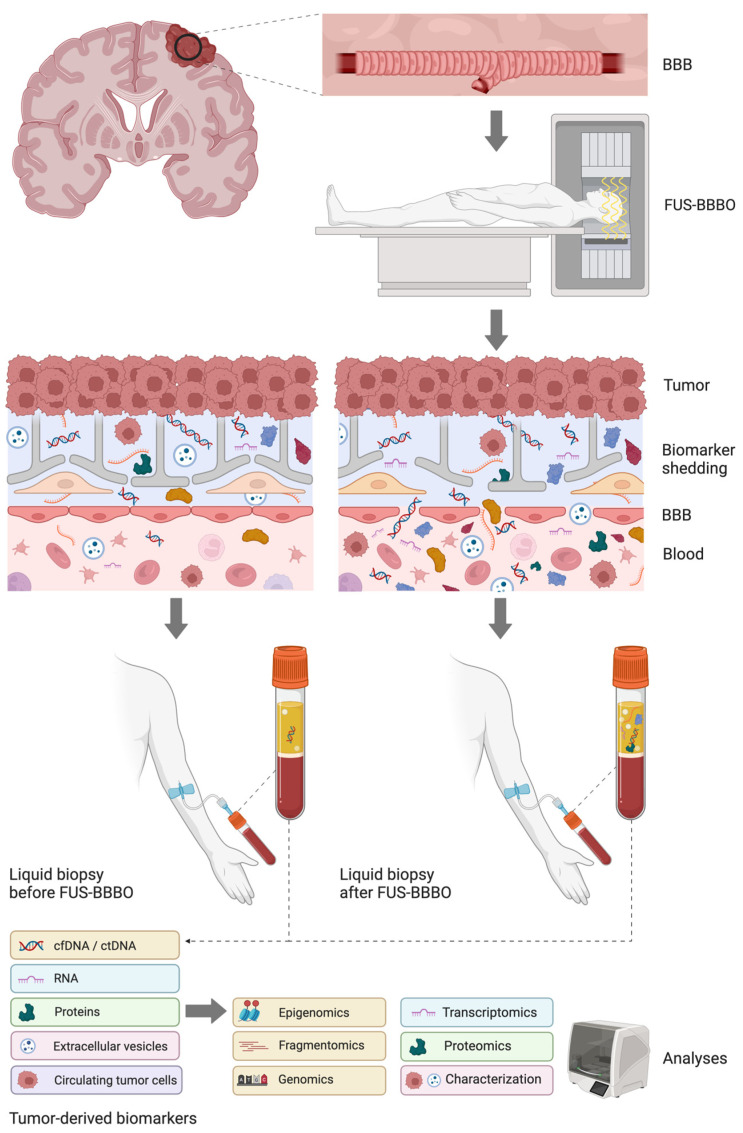
Focused ultrasound-mediated blood–brain barrier opening (FUS-BBBO) and liquid biopsy in brain tumors. The blood–brain barrier (BBB) hampers the transport of biomarkers shed by the brain tumor in the bloodstream; FUS-BBBO temporarily increases the permeability of the BBB in the tumor vasculature allowing tumor-derived biomarkers to enter the bloodstream. Blood sample collection shortly after FUS-BBBO (FUS-BBBO-enhanced liquid biopsy) could improve the blood levels of tumor-derived biomarkers, including cell-free DNA (cfDNA), circulating tumor DNA (ctDNA), RNA, proteins, extracellular vesicles and circulating tumor cells (CTC). Biomarker enrichment by FUS-BBBO could be confirmed by comparing biomarker levels in blood samples drawn before and after FUS-BBBO. The following analyses can be applied to the abovementioned analytes to identify tumor-associated signatures: epigenomic and genomic profiling (e.g., methylation pattern, respectively, point mutations and copy number variations) or fragmentomic analyses (fragment size) on cfDNA, transcriptomic analyses (RNA), proteomic analyses (protein expression and posttranslational modifications) or quantification and characterization of extracellular vesicles and circulating tumor cells. Created with https://BioRender.com, accessed on 26 January 2024.

A promising strategy to enhance the blood-based brain tumor biomarker detectability in patients is through focused ultrasound-mediated BBB opening, also known as FUS-BBBO (Figure 1). Focused ultrasound, in combination with microbubbles, can temporarily and locally open the BBB [16]. The intravenously injected microbubbles will start oscillating when they pass through the focused ultrasound field. Parameters such as the ultrasound frequency, acoustic pressure microbubble diameter, pulse-repetition frequency and burst duration impact microbubble oscillations. A simplified manner to describe the impact of ultrasound frequency and acoustic pressure on microbubble behavior is the mechanical index (MI). The MI is determined by dividing the negative acoustic pressure by the square root of the ultrasound frequency [17].

At a low MI, microbubbles display a stable oscillation of amplitudes less than the typical capillary diameter. The repeated expansion and compression of microbubbles when exposed to low acoustic pressures is known as stable or non-inertial cavitation. The expansion of microbubbles near the vessel wall may push apart the endothelial lining. Conversely, the shrinkage of microbubbles can lead to invaginations in the vascular lining, potentially causing the opening of tight junctions through push–pull mechanisms. Additionally, rapid expansion and contraction characteristics of microbubbles in an ultrasonic field have been found to generate micro-streams capable of inducing high shear stresses up to several thousand Pascals, thereby compromising the integrity of the endothelial cell membrane permeability. Furthermore, by absorbing US energy, pressure gradients are generated and acoustic radiation forces displace microbubbles in the direction of the US wave generation. This causes microbubbles to strongly push the endothelium, thereby increasing vascular permeability [18,19,20,21]. Although the exact mechanism of FUS-BBBO is under active research and most likely multifactorial, several biological processes are triggered, including the disruption of endothelial cell tight junctions, potentiation of transcytosis [22], and quantitative and qualitative downregulation of ATP-binding Cassette (ABC) drug transporter family member function such as P-glycoprotein (P-gp) functionality [23]. Altogether, this increases the BBB permeability, which could potentially increase the concentration of biomarkers in the blood [24].

Importantly, if the MI surpasses a specific threshold, microbubbles oscillate at amplitudes exceeding the mechanical limitations of their shell structure, leading ultimately to shell rupture and subsequent violent collapse of the resulting free-gas bubbles, known as inertial cavitation. This can result in fragmentation of microbubbles, creating high temperatures and pressures in the vicinity. This process typically involves shock waves and micro-jet formation, both of which can perforate cell membranes and increase vascular permeability through bio-physical effects [19,20]. These effects produced by inertial cavitation can cause micro-damage to the vessel walls, resulting in the extravasation of erythrocytes [25]. 

Whether FUS-BBBO can safely lead to increased levels of brain tumor biomarkers in circulating blood is currently an area of ongoing research. In this systematic review, we aim to comprehensively present current knowledge on FUS-BBBO-enhanced liquid biopsy in both animal models and patients with primary brain tumors.

## 2. Materials and Methods

A systematic search of PubMed and Embase was performed (final search 20 October 2023) with the use of MeSH and Emtree terms, respectively, for primary brain tumors, biomarkers, ultrasound and their synonyms (Supplementary Table S1). The Preferred Reporting Items for Systematic Reviews and Meta-Analyses (PRISMA) guidelines were followed [26]. This study has not been registered. 

After eliminating duplicate and non-English articles, two authors (AB, AEI) conducted a stepwise selection process based on the titles, abstracts and full texts. Articles were included if: (1) the study concerned original research; (2) the disease under examination concerned primary brain tumors; (3) FUS-BBBO was performed; (4) the influence of FUS-BBBO on tumor-associated biomarker levels was investigated.

In addition to animal models, patient and FUS-BBBO procedure characteristics, we extracted details regarding the FUS-BBBO devices; FUS parameters; and liquid biopsy details, including sample type, sample volume, sampling method and sampling time; the type of assessed biomarkers and corresponding analytical techniques used to detect tumor biomarkers levels in blood; and complications of FUS-BBBO. We compared the MI of the experiments and the resulting biomarker fold change and complications.

Results are presented separately and descriptively for preclinical and clinical studies. Study methods and study results are reported separately. For reporting ultrasound parameters, we followed the guidelines of Padilla and ter Haar [27]. To enable the comparison of the ultrasound settings across experiments, we calculated the MI for each experiment.

Two different tools were used to evaluate the preclinical and clinical studies’ risk of bias by three authors (AB, AEI, HV). The SYRCLE RoB tool was selected for preclinical studies, and the NIH Quality Assessment Tool was selected to assess studies without a control group [28,29]. During the evaluation, relevant information contained in the tools was identified and assessed, while any unclear aspects were noted.

## 3. Results

**Figure 2:**
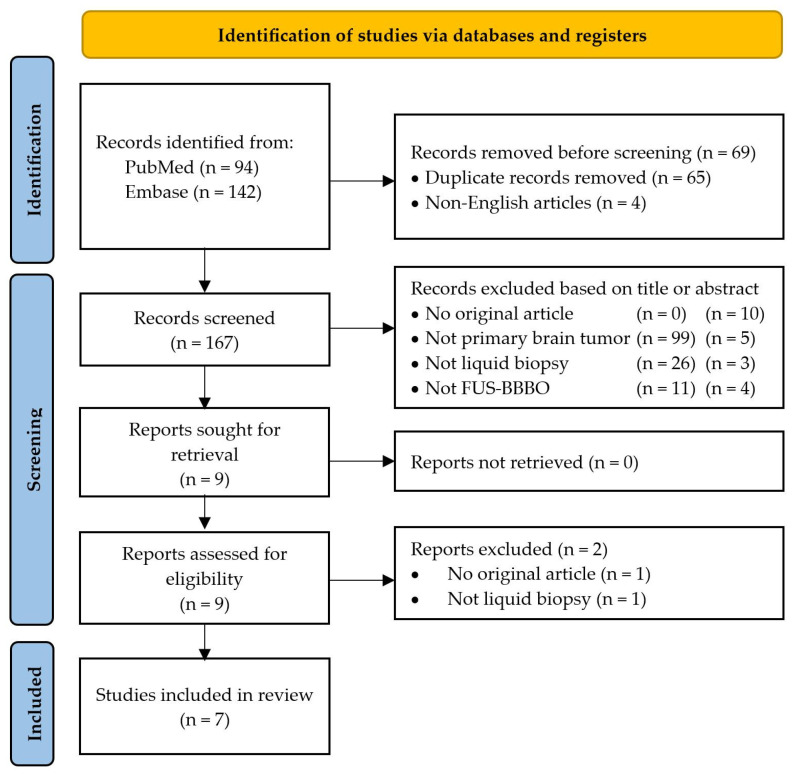
PRISMA flow diagram for step-by-step study selection. Abbreviations: FUS-BBBO = focused ultrasound-mediated blood–brain barrier opening.

Our search yielded 236 records, as depicted in Figure 2 of the PRISMA 2020 flow diagram. Ultimately, seven records met the eligibility criteria, comprising two clinical and five preclinical studies [24,30,31,32,33,34,35]. The included studies were published between April 2018 and September 2023.

### 3.1. Preclinical Studies

Five studies from three different research groups investigated FUS-BBBO in animal models (Supplementary Table S2). The risk of bias in all preclinical studies was high (Supplementary Table S3). Four of these studies utilized mouse models [24,30,32,34], whereas one of these four studies also employed a porcine model [34]. A single study used a rat model [31]. The brain tumor models were created through intracranial injection of glioma cell lines, including 9L [31], GL261 [24,32], PF8 [30] and U87 [24,34]. Experiments on healthy control animals reported in the articles were considered in this review. 

Several preclinical FUS devices/transducers were used for pulsed FUS with the frequency ranging from 0.65 MHz to 3.3 MHz. Single [24,30,32,34], double [30,34] or five sessions [31] were given to treat one [30,31,32] or multiple [24,34] locations in the brain. FUS was combined with microbubbles given with a bolus injection that were either in-house developed [24,31,32] or commercially available [30,34], i.e., Definity^®^ (Lantheus Medical Imaging, North Billerica, MA, USA) and Lumason^®^ (Bracco Diagnostics Inc., Monroe Township, NJ, USA). 

Various (tumor) biomarkers for liquid biopsy were investigated: mRNA [24,32]; cfDNA [30], including ctDNA [34]; and proteins [31]. To detect these biomarkers in the blood, several strategies were employed, such as ELISA [31], qPCR [24,32], ddPCR [30,34] and Qubit High Sensitivity dsDNA assay [30] or Qubit Fluorometric Quantitation [34]. In small animal studies, liquid biopsy results were compared with a control group. Data were lacking on the comparison between animals undergoing FUS-BBBO and controls regarding randomization [24,30,31], group size [30] and tumor size [24,30,31,34]. In contrast, only in the porcine model [34] were biomarker levels in the pre- and post-FUS-BBBO blood samples compared. 

**Figure 3:**
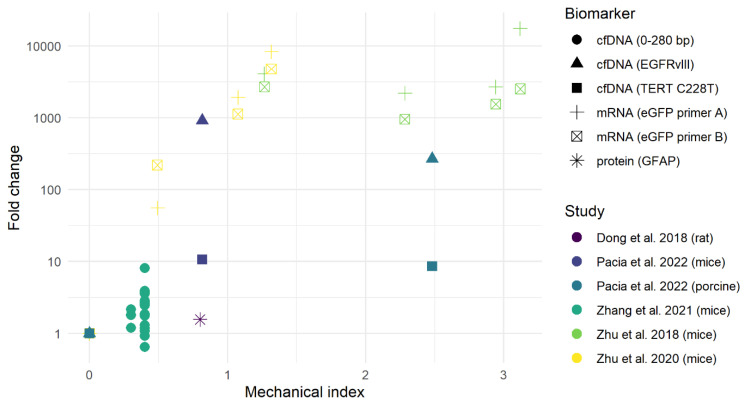
Mechanical index vs. fold change in biomarker level for animals receiving FUS-BBBO vs. controls, or the change from pre- to post-FUS-BBBO [24,30,31,32,34]. Abbreviations: cfDNA = circulating free DNA; bp = base pair; EGFRvIII = epidermal growth factor receptor variant III; TERT C228T = telomerase reverse transcriptase promotor mutation C228T; mRNA = messenger RNA; eGFP = enhanced green fluorescent protein; GFAP = glial fibrillary acidic protein.

The experiments applied MIs, resulting in both stable cavitation [30,31] and inertial cavitation [24,32,34] of the microbubbles. Studies investigated the effect of increasing peak negative pressure, single or multiple FUS-BBBO sessions and the timing of blood sampling after FUS-BBBO. All five studies showed either stable or increased levels of biomarkers with FUS-BBBO compared to controls (Table 1). The release of biomarkers (mRNA, cfDNA and proteins) varied for different MIs (Figure 3). Biomarker levels in the FUS-BBBO group with inertial cavitation were 100–20,000-fold higher compared to controls [24,32,34]. Stable cavitation increased biomarker levels from controls to the FUS-BBBO group by 1.5–8.1-fold [30,31]. One outlier can be identified in Figure 3, i.e., the results in the porcine model [34]. Likely the MI is overestimated in this model since the calculation was based on hydrophone measurements with an ex vivo porcine skull.

One study using stable cavitation tested the hypothesis that the release of biomarkers after FUS-BBBO is time-dependent [30]. Repeated blood sampling after FUS-BBBO at different time points (2 min, 15 min, 30 min, 45 min, 60 min and 24 h) indicated that the amount of cfDNA in the blood started to increase 15 min after FUS-BBBO and remained increased at the 60-min measure point. Twenty-four hours after FUS-BBBO, the biomarker level had returned to baseline. This was shown in both control and glioma mice. The impact of tumor volume on biomarker release was compared for FUS-BBBO procedures at day 7 and day 20 post intracranial injection of tumor cells; the fold change in cfDNA level in the blood increased from 2.9 to 8.1, respectively. Of note, this study reported cfDNA levels without mentioning to what degree the cfDNA was tumor-specific. The study used fixed settings for microbubble type, manufacturer and dose in the experiments [30].

The detection sensitivity of tumor-specific mutations found in the ctDNA improved after FUS-BBBO with inertial cavitation [34]. The detection sensitivity of epidermal growth factor receptor variant III (EGFRvIII) was improved by FUS-BBBO from 7% to 65%, and the telomerase reverse transcriptase (TERT) promotor mutation C228T from 14% to 46% in the mouse glioblastoma model. This was confirmed in the porcine glioblastoma model, where the diagnostic sensitivity for EGFRvIII improved by FUS-BBBO from 29% to 100%, and for TERT C228T from 43% to 71%.

#### Complications

**Figure 4:**
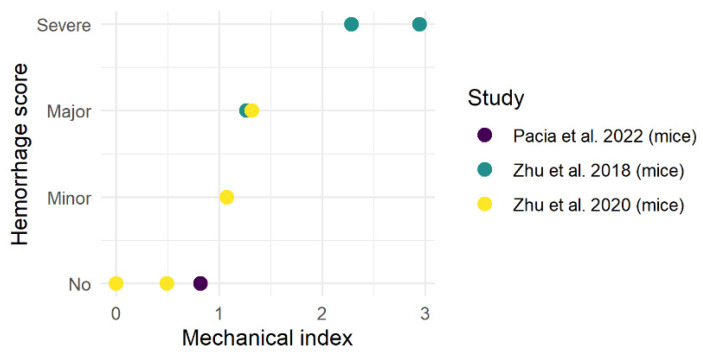
The severity of hemorrhaging as a function of the applied mechanical index. Hemorrhage is categorized as having no more damage than control animals without FUS-BBBO, minor damage (5–8 times more damage than control), major damage (10–14 times more damage than control) and severe damage (>15 times more damage than control). Data were retrieved from [24,32,34].

Tissue damage was assessed by comparing Hematoxylin and Eosin (H&E) staining [24,32,34] and terminal deoxynucleotidyl transferase dUTP nick end labeling (TUNEL) staining [34] of ex vivo (tumor) slices from treated and control animals. TUNEL staining did not indicate a difference in apoptotic cells between the tumor and brain parenchyma [34]. H&E staining was used in three studies to assess the extravasation of red blood cells into the brain parenchyma, i.e., (micro-) hemorrhaging [21,22,31]. The occurrence and severity of hemorrhaging depended on the applied ultrasound parameters. Hemorrhaging only occurred in experiments that applied MIs, which induced inertial cavitation. In Figure 4, the MI was compared to the severity of hemorrhaging. We categorized the severity of hemorrhaging as having no (same as control), minor (5–9 times more than control), major (10–14 times more than control) or severe (>15 times more than control) hemorrhaging in the H&E slices. Below an MI of approximately 1.0, there was no evidence of micro-hemorrhaging, whereas above an MI of approximately 1.0, extravasation of red blood cells is evidenced.

### 3.2. Clinical Studies

Two clinical studies assessed FUS-BBBO assisted liquid biopsy in fourteen patients (age 52.4 ± 13.0 years), where 13 had received the diagnosis of glioblastoma, and one patient had a diffuse high-grade glioma, not further specified (Table 2) [33,35]. Most tumors were IDH-1 wildtype, and only one patient had an IDH mutation (R132H); the latter tumor would now be (re-)classified as an astrocytoma, IDH-mutant, WHO grade 4. In one study, FUS-BBBO was combined with temozolomide delivery [33], whereas the other study solely focused on FUS-BBBO [35]. The risk of bias in both clinical studies was low (Supplementary Table S4).

Two different FUS-BBBO devices were used: a neuronavigation-guided 650 kHz FUS device (Imasonic, Voray-sur-l’Ognon, France), and the 220 kHz MRI-guided Exablate Neuro Type 2 (Insightec, Haifa, Israel). Patients received either one treatment with a small target volume, 0.13 cm^3^ [35], or two to six monthly treatments combined with temozolomide with larger target volumes, ranging from 2.48 to 21.16 cm^3^ [33]. In all patients, Definity^®^ (Lantheus Medical Imaging, North Billerica, MA, USA) microbubbles were administered to induce stable microbubble cavitation with FUS. Blood samples pre- and post-FUS-BBBO were compared. 

Various (tumor) biomarkers for the liquid biopsy were investigated: cfDNA [33,35], including ctDNA [35]; extracellular vesicles [33]; and proteins [33]. To detect these biomarkers in the blood, several methods were used, such as ELISA [33], ddPCR [33,35] and a personalized tumor-informed ctDNA assay [35]. Furthermore, the methylation signature of blood was investigated by the Illumina Methylation EPIC 850k array [33].

FUS-BBBO treatments resulted in either stable or increased concentration of (tumor) biomarkers in the blood (Table 3) [33,35]. Yuan et al. [35] found that FUS-BBBO increased the concentration of mononucleosome cfDNA fragments (120–280 bp) in four out of five patients. Samples of the blood were taken at different time points after FUS, e.g., 5, 10 and 30 min. The time of peak cfDNA and ctDNA concentrations in the blood varied between patients, from 10 to 30 min after FUS. The maximum increase was 1.6-fold for the mononucleosome cell-free DNA (cfDNA) fragments (120–280 bp), 1.9-fold for patient-specific tumor variant ctDNA level, and 5.6-fold for the TERT mutation ctDNA level [35].

Meng et al. [33] took blood samples with a median sampling time of 34 min post-FUS. Several biomarker levels increased after FUS-BBBO, including plasma cfDNA concentration (2.6 ± 1.2-fold), neuron-derived extracellular vesicles (3.2 ± 1.9-fold) and brain-specific protein S100b (1.4 ± 0.2-fold). The cfDNA methylation signature was different for pre- and post-FUS-BBBO. In a single patient with an R132H IDH mutation, the mutant copies in the plasma were increased two- to three-fold post-FUS [33].

The size of the biomarker, ranging from cfDNA to 50 Dalton proteins and extracellular vesicles, was not associated with the fold change in biomarker concentration of the blood [33,35].

#### Complications

Yuan et al. [35] assessed tissue damage by FUS-BBBO, where the tumor was resected within 1.7 ± 0.4 h after FUS-BBBO. H&E staining of the sonicated and non-sonicated brain tumor tissue did not show clear evidence of tissue damage in five patients [35]. Furthermore, transcriptomic analysis of the resected tissue of three patients did not show evident inflammatory/ immune response within 1.7 ± 0.4 h after FUS-BBBO.

## 4. Discussion

In this systematic review, we aimed to present the current knowledge about tumor biomarker release and potential toxicity following FUS-BBBO. This is a relatively new research field, which is reflected in the low number of papers that could be included. The available studies suggest that focused ultrasound in combination with microbubbles can elevate brain- and brain-tumor-derived biomarker levels in the plasma across different animal glioma models and patients with high-grade brain tumors [24,30,31,32,33,34,35]. Blood samples pre- and post-FUS-BBBO can be compared to obtain a tumor-specific signature. However, depending on the applied US parameters, FUS-BBBO can induce either stable or inertial cavitation of the microbubbles, which have very different effects on the vasculature. Both applications were included in this systematic review.

Most preclinical studies investigating FUS-BBBO-enhanced liquid biopsy used inertial cavitation (MI > 0.8), which resulted in 100–20,000-fold increases in biomarker concentrations at the expense of local hemorrhaging and BBB damage (Figure 3 and Figure 4) [24,32,34]. Other studies applied stable cavitation (MI < 0.8) without evidence of tissue damage and achieved a 1.5–8.1-fold increase in biomarker concentration [30,31]. The MI threshold at which the transition from stable to inertial cavitation occurs varies for different microbubbles and is approximately at the 0.6 MI and 0.8 MI threshold for Lumason^®^ and Definity^®^ microbubbles, respectively [36]. In patients, only FUS-BBBO with stable cavitation has been applied to enhance liquid biopsy without the risk of hemorrhages [33,35]. However, it should be further investigated and debated whether inducing micro-hemorrhages in the human brain is acceptable to optimize the yield of FUS-BBBO-enhanced liquid biopsy, as long as patient safety is maintained. In addition to the MI, a wide range of variables affect the safety profile of FUS-BBBO, including FUS parameters (e.g., pulse-repetition frequency and burst duration impact), microbubble characteristics (e.g., size, composition) and dose and the patient’s microvascular anatomy [37].

Upon stable microbubble cavitation, the release of biomarkers was time-dependent, as was shown by the varying biomarker concentrations over time in both mice [30] and patients [35]. The highest biomarker concentrations occurred between 10 min and 1 h after FUS-BBBO, but this was highly animal- and patient-specific [30,35]. Future clinical trials testing FUS-BBBO in brain diseases should incorporate various blood sampling intervals after FUS-BBBO to optimize timing. We hypothesize that the time-dependent release of biomarkers after FUS-BBBO also depends on biomarkers’ characteristics, such as their size and half-life in blood, where BBBO closure kinetics probably play a role. This should be investigated in future preclinical studies. 

Except for Yuan et al. [35], studies seem to indicate that some degree of inflammatory response follows FUS-mediated increases in BBB permeability; however, this is reported in tissue samples taken >6 h post-FUS-BBBO, compared to the tissue sample taken at 1.7 h post-BBBO in Yuan et al. [35]. The degree of inflammatory response following FUS-BBBO is reported to be variable and seems to depend on BBB permeability, as summarized by McMahon et al. [37].

### 4.1. Ongoing and Future Clinical Trials

It is crucial to exercise caution when introducing novel diagnostic tools, including FUS-BBBO. These tools are designed to assess a patient’s medical condition or disease progression and, ultimately, to guide subsequent patient management. Future studies are needed to fully evaluate the diagnostic accuracy and therapeutic decisive relevance of FUS-BBBO-assisted liquid biopsy before its integration into clinical practice.

Several ongoing clinical trials investigating FUS-BBBO as a drug delivery technique include biomarker level changes as an exploratory endpoint (e.g., NCT04667715, NCT05293197 and NCT04528680). LIBERATE (NCT05383872) [38] is studying the use of FUS-BBBO with the Exablate Neuro Type 2 device (Insightec, Haifa, Israel). The primary endpoint in LIBERATE is defined as the fold-change between the cfDNA level in the blood sample taken one-hour post-FUS-BBBO and the level pre-FUS-BBBO. In the secondary analyses, blood-derived tumor biomarkers (sampled 1 h after FUS-BBBO) will be compared to neurosurgically obtained tissue. Explored outcomes will include the detection sensitivity of somatic mutations in cfDNA in blood samples before and after BBBO. Furthermore, they will investigate the optimal time for cfDNA yield by collecting post-BBBO blood samples at 30, 60, 120 and 180 min post-BBBO. The correlation of imaging biomarkers with post-BBBO biomarker samples will be examined [38]. Another upcoming study will be conducted with the Cordance device [39] to examine FUS-BBBO in patients with recurrent glioblastoma. The study will compare the concentration of cfDNA in plasma before and after the procedure.

Other focused ultrasound techniques have also been found to enhance brain biomarkers in liquid biopsy. For example, focused ultrasound hyperthermia enhanced the release of extracellular vesicles in mice [40]. Focused ultrasound thermal ablation is currently being investigated as a method to enhance the concentration of ctDNA in the blood of glioma patients (NCT04940507, n = 50).

### 4.2. Limitations

The included studies and our analyses had several limitations beyond the limited number of studies and small sample sizes. As discussed previously, there was much variation in animal models, tumor models, stable vs. inertial cavitation, microbubbles, biomarkers, sample methods and timing, which impacted the study results. Furthermore, brain tumor-specific biomarkers of humans are released into a larger total blood volume after FUS-mediated BBB opening compared to mice and pigs. On average, humans have a total blood volume of 5 L, while piglets have 600 mL and mice have 1.5 mL. This is important to note because the sensitivity of analysis techniques detecting biomarkers depends on the number of biomarkers that are released into the respective blood volume. Because of the low total blood volume in mice, repeated blood sampling in sufficient quantities for the employed analysis methods after FUS-BBBO was not feasible in these animals. Repeated blood sampling is important to evaluate the reproducibility of this technique in humans and to determine the optimal time for blood collection after FUS treatment. Timing is particularly important as the half-life and clearance kinetics of biomarkers have to be taken into consideration. Furthermore, differences in biomarker levels between different animals/humans/time intervals can be influenced by intrinsic tumor shedding potential, BBB constitution, BBB-closure kinetics and biomarker clearance kinetics. Lastly, the sensitivity of detection methods also depends on the background signal from healthy circulating nucleic acids. In patient-derived xenograft (PDX) models, the background signal is, per definition, non-human, which can facilitate the detection of human tumor-derived material in the blood, thereby exaggerating sensitivity. Both studies of Zhu et al. [24,32] investigated the exogenous biomarker eGFP, which is naturally not present in primary brain tumors and is derived from a jellyfish. The detection of eGFP in plasma is, therefore, easier compared to brain tumor-derived biomarkers. Several studies only quantified the total level of cfDNA and did not explore the origin or further characteristics of the cfDNA that was released into the bloodstream post-sonication [30,33]. 

### 4.3. Risk of Bias

In general, the risk of bias in the preclinical studies was high. This is mostly due to missing data regarding critical aspects of study design. Clinical studies were found to have a low risk of bias, but they were conducted with a limited number of patients. Therefore, it remains uncertain whether the results obtained from these studies can be generalized. Thus, follow-up research is needed to validate and expand the existing knowledge.

### 4.4. Future Prospects

The potential of FUS-BBBO induced by stable microbubble cavitation to optimally increase biomarker levels without inducing toxicity should be investigated further in both preclinical and clinical trials. FUS-BBBO has the potential to open the BBB of the entire tumor, whereas brain tumor biomarkers found in the blood without FUS-BBBO may only represent a small portion of the tumor with a leaky BBB.

Future research should optimize settings to maximize the amplification of marker levels in patients without inducing tissue damage. Biomarker detection can be further improved with the use of technical advancements, including improved sequencing depth, corrective algorithms [41] and the implementation of artificial intelligence (AI). Afterward, structured clinical studies with rigorous evaluation of diagnostic test accuracy should follow. 

Further studies are needed to assess the potential risks of FUS-BBBO-enhanced liquid biopsy. Theoretically, the temporarily increased permeability of the BBB might lead to metastasis. Presently, there are no reports of metastasis after FUS-BBBO in the literature. Clinical studies have mainly focused on short-term outcomes, overlooking this potential risk. On the other hand, extracranial metastases from primary brain tumors (including tumors with a partly disrupted BBB) are rare, which may be due in part to the short survival time of patients, intrinsic tumor cell characteristics and the unfavorable microenvironment for brain tumor cells outside the CNS.

## 5. Conclusions

FUS-BBBO holds significant potential to safely, feasibly and minimally invasively increase brain tumor-specific biomarker levels in the bloodstream. Current evidence is limited to five in vivo studies utilizing mouse, rat and pig glioblastoma models and two clinical studies, including fourteen patients with high-grade glioma. According to the findings of this review, FUS-BBBO based on stable cavitation can safely increase a variety of tumor-derived biomarkers in the bloodstream of patients with brain tumors, including circulating nucleic acids, extracellular vesicles and proteins. Tissue damage as a result of inertial microbubble cavitation was found at an MI above 1.0. In patient studies with stable microbubble cavitation, the FUS-BBBO-mediated increase in biomarkers was 1.5 to 5.6-fold; further methodological studies are needed to improve the effect of FUS-BBBO on biomarker levels. Overall, FUS-BBBO-mediated liquid biopsy is still in its infancy. Nevertheless, it shows promise and may fundamentally alter the diagnostic process for patients with primary brain tumors, facilitating more timely identification and personalized treatment. 

## Figures and Tables

**Table 1:** Overview of biomarkers used for liquid biopsy.

Circulating Factor	Advantage	Disadvantage
Circulating tumor cell (CTC)	Highly specific Different levels (DNA, RNA, protein)	Elaborate isolation techniques No standard method (few studies for glioblastoma)
Protein	Expression directly related to cellular functions	Nonspecific Complex background (immuno, physiological)
Extracellular vesicles (EV)	Better source nucleic acids ctDNA Able to cross BBB (easy detection)	High background nontumor EVs No standard method of isolation
ctDNA	Highly specific Allows molecular classification Known isolation methods Higher circulating levels than CTC	Low sensitivity of detection (0.1–0.5% in blood) Short half-life (<1.5 h) Only represent subpopulation tumor cells
RNA	Higher sensitivity detection May aid prognosis/monitoring	Nonspecific No standard method of isolation

**Table 2:** Characteristics of the included patients, their tumors and the FUS procedures. Tumor type was converted into (likely) WHO2021 diagnosis wherever possible. Abbreviations: IDH = isocitrate dehydrogenase, TERT = telomerase reverse transcriptase; MGMT = O-6-methylguanine-DNA methyltransferase; FUS = Focused ultrasound; MI = Mechanical index; # = number of FUS procedures.

Study	Patient			Tumor Type			FUS Procedures		
	ID	Age	Sex	Type	TERT	MGMT Promotor	#	Sonication Volume (cm^3^)	Estimated MI
[35]	G01	73	M	Glioblastoma, IDH-wildtype	C228T	n.r.	1	0.13	0.58
	G02	58	M	Glioblastoma, IDH-wildtype	C250T	n.r.	1	0.13	0.38
	G03	66	F	Glioblastoma, IDH-wildtype	C228T	n.r.	1	0.13	0.8
	G04	66	M	Glioblastoma, IDH-wildtype	C250T	n.r.	1	0.13	0.66
	G05	36	M	High-grade glioma NOS, IDH-wildtype	Wild type	n.r.	1	0.13	0.5
[33]	P1	49	F	Glioblastoma, IDH-wildtype	n.r.	NA	4	3.75	n.r.
	P2	52	M	Glioblastoma, IDH-wildtype	n.r.	Methylated	6	4.28	n.r.
	P3	56	F	Glioblastoma, IDH-wildtype	n.r.	Unmethylated	4	2.48	n.r.
	P4	35	M	Glioblastoma, IDH-wildtype	n.r.	NA	3	4.97	n.r.
	P5	56	F	Glioblastoma, IDH-wildtype	n.r.	Unmethylated	4	6.88	n.r.
	P6	42	F	Astrocytoma, IDH-R132-H-mutant, grade 4	n.r.	NA	6	8.13	n.r.
	P7	40	F	Glioblastoma, IDH-wildtype	n.r.	Unmethylated	4	9.08	n.r.
	P8	36	F	Glioblastoma, IDH-wildtype	n.r.	Unmethylated	5	21.16	n.r.
	P9	68	M	Glioblastoma, IDH-wildtype	n.r.	Methylated	2	5.55	n.r.

**Table 3:** Fold change in biomarker level in patients with high-grade glioma (n = 14; including 13 patients with glioblastoma) comparing samples pre- and post-FUS-BBBO. Data retrieved from [32,34]. Abbreviations: ctDNA = circulating tumor DNA; TERT = telomerase reverse transcriptase; IDH = isocitrate dehydrogenase; cfDNA = circulating free DNA; bp = base pair; NCAM = neural cell adhesion molecule; L1CAM = L1 cell adhesion molecule.

Biomarker	Specific Target	n	Fold Change	Patients with Fold Increase (%)	Data From
ctDNA		5	1.5 ± 0.4	60	[35]
	Mutant copies TERT C228T/C250T	2	4.3 and 5.6	50	[35]
	Mutant copies IDH1-R132H	1	2 to 3	100	[33]
cfDNA: fragment size selection		9	2.6 ± 1.2	100	[33]
	0–280 bp	9	3.5	100	[33]
	120–280 bp	5	1.3 ± 0.2	80	[35]
Protein	S100b	9	1.4 ± 0.2	89	[33]
Extracellular vesicles	NCAM and L1CAM	9	3.2 ± 1.9	100	[33]

## Data Availability

Data are contained within the article.

## Supplementary Materials

The following supporting information can be downloaded at https://www.mdpi.com/article/10.3390/cancers16081576/s1, Table S1: Search string in Pubmed and Embase; Table S2: Characteristics and results of the preclinical studies included in the systematic review; Table S3: Risk of bias of the preclinical studies assessed with SYRCLE’s (SYstematic Review Centre for Laboratory animal Experimentation) RoB (Risk of Bias) tool (released in 2014); Table S4: Risk of bias of the clinical studies assessed with the National Institutes of Health (NIH) quality assessment tool for before-after (Pre-Post) study with no control group. References [24,30,31,32,34,35,42] are cited in the Supplementary Materials.

## References

[1] Ostrom Q.T., Gittleman H., Truitt G., Boscia A., Kruchko C., Barnholtz-Sloan J.S. (2018). CBTRUS Statistical Report: Primary Brain and Other Central Nervous System Tumors Diagnosed in the United States in 2011–2015. Neuro Oncol..

[2] Miller K.D., Ostrom Q.T., Kruchko C., Patil N., Tihan T., Cioffi G., Fuchs H.E., Waite K.A., Jemal A., Siegel R.L. (2021). Brain and Other Central Nervous System Tumor Statistics, 2021. CA Cancer J. Clin..

[3] Jusue-Torres I., Hulbert A., Barton K., Melian E., Anderson D.E., Quinones-Hinojosa A., Prabhu Vikram C. (2022). Survival Benefit of Concomitant Chemoradiation in Adult Supratentorial Primary Glioblastoma. A Propensity Score Weighted Population-Based Analysis. J. Neurosurg. Sci..

[4] Li B.K., Al-Karmi S., Huang A., Bouffet E. (2020). Pediatric Embryonal Brain Tumors in the Molecular Era. Expert Rev. Mol. Diagn..

[5] Pollack I.F., Agnihotri S., Broniscer A. (2019). Childhood Brain Tumors: Current Management, Biological Insights, and Future Directions. J. Neurosurg. Pediatr..

[6] Pardridge W.M. (2005). The Blood-Brain Barrier: Bottleneck in Brain Drug Development. NeuroRx.

[7] Chughtai K.A., Nemer O.P., Kessler A.T., Bhatt A.A. (2019). Post-Operative Complications of Craniotomy and Craniectomy. Emerg. Radiol..

[8] Kickingereder P., Willeit P., Simon T., Ruge M.I. (2013). Diagnostic Value and Safety of Stereotactic Biopsy for Brainstem Tumors: A Systematic Review and Meta-Analysis of 1480 Cases. Neurosurgery.

[9] Draaisma K., Chatzipli A., Taphoorn M., Kerkhof M., Weyerbrock A., Sanson M., Hoeben A., Lukacova S., Lombardi G., Leenstra S. (2019). Molecular Evolution of IDH Wild-Type Glioblastomas Treated With Standard of Care Affects Survival and Design of Precision Medicine Trials: A Report From the EORTC 1542 Study. J. Clin. Oncol..

[10] Warton K., Mahon K.L., Samimi G. (2016). Methylated Circulating Tumor DNA in Blood: Power in Cancer Prognosis and Response. Endocr. Relat. Cancer.

[11] Berzero G., Pieri V., Mortini P., Filippi M., Finocchiaro G. (2023). The Coming of Age of Liquid Biopsy in Neuro-Oncology. Brain.

[12] Le Rhun E., Seoane J., Salzet M., Soffietti R., Weller M. (2020). Liquid Biopsies for Diagnosing and Monitoring Primary Tumors of the Central Nervous System. Cancer Lett..

[13] Bettegowda C., Sausen M., Leary R.J., Kinde I., Wang Y., Agrawal N., Bartlett B.R., Wang H., Luber B., Alani R.M. (2014). Detection of Circulating Tumor DNA in Early-and Late-Stage Human Malignancies. Sci. Transl. Med..

[14] Fontanilles M., Duran-Peña A., Idbaih A. (2018). Liquid Biopsy in Primary Brain Tumors: Looking for Stardust!. Curr. Neurol. Neurosci. Rep..

[15] Eibl R.H., Schneemann M. (2021). Liquid Biopsy and Primary Brain Tumors. Cancers.

[16] Hynynen K., McDannold N., Vykhodtseva N., Jolesz F.A. (2001). Noninvasive MR Imaging-Guided Focal Opening of the Blood-Brain Barrier in Rabbits. Radiology.

[17] Chen K.T., Wei K.C., Liu H.L. (2019). Theranostic Strategy of Focused Ultrasound Induced Blood-Brain Barrier Opening for CNS Disease Treatment. Front. Pharmacol..

[18] Van Bavel E. (2007). Effects of Shear Stress on Endothelial Cells: Possible Relevance for Ultrasound Applications. Prog. Biophys. Mol. Biol..

[19] Lentacker I., De Cock I., Deckers R., De Smedt S.C., Moonen C.T.W. (2014). Understanding Ultrasound Induced Sonoporation: Definitions and Underlying Mechanisms. Adv. Drug Deliv. Rev..

[20] Dasgupta A., Liu M., Ojha T., Storm G., Kiessling F., Lammers T. (2016). Ultrasound-Mediated Drug Delivery to the Brain: Principles, Progress and Prospects. Drug Discov. Today Technol..

[21] Bunevicius A., McDannold N.J., Golby A.J. (2020). Focused Ultrasound Strategies for Brain Tumor Therapy. Oper. Neurosurg..

[22] Sheikov N., McDannold N., Vykhodtseva N., Jolesz F., Hynynen K. (2004). Cellular Mechanisms of the Blood-Brain Barrier Opening Induced by Ultrasound in Presence of Microbubbles. Ultrasound Med. Biol..

[23] Cho H.S., Lee H.Y., Han M., Choi J.R., Ahn S., Lee T., Chang Y., Park J. (2016). Localized Down-Regulation of P-Glycoprotein by Focused Ultrasound and Microbubbles Induced Blood-Brain Barrier Disruption in Rat Brain. Sci. Rep..

[24] Zhu L., Cheng G., Ye D., Nazeri A., Yue Y., Liu W., Wang X., Dunn G.P., Petti A.A., Leuthardt E.C. (2018). Focused Ultrasound-Enabled Brain Tumor Liquid Biopsy. Sci. Rep..

[25] McDannold N., Vykhodtseva N., Hynynen K. (2006). Targeted Disruption of the Blood-Brain Barrier with Focused Ultrasound: Association with Cavitation Activity. Phys. Med. Biol..

[26] Page M.J., McKenzie J.E., Bossuyt P.M., Boutron I., Hoffmann T.C., Mulrow C.D., Shamseer L., Tetzlaff J.M., Akl E.A., Brennan S.E. (2021). The PRISMA 2020 Statement: An Updated Guideline for Reporting Systematic Reviews. BMJ.

[27] Padilla F., ter Haar G. (2022). Recommendations for Reporting Therapeutic Ultrasound Treatment Parameters. Ultrasound Med. Biol..

[28] Hooijmans C.R., Rovers M.M., de Vries R.B., Leenaars M., Ritskes-Hoitinga M., Langendam M.W. (2014). SYRCLE’s Risk of Bias Tool for Animal Studies. BMC Med. Res. Methodol..

[29] NHLBI Quality Assessment Tool for Before-After (Pre-Post) Studies with No Control Group. https://www.nhlbi.nih.gov/health-topics/study-quality-assessment-tools.

[30] Zhang D.Y., Gould A., Happ H.C., Youngblood M.W., Dmello C., Kang S.J., Canney M., Stupp R., Carvill G.L., Sonabend A.M. (2021). Ultrasound-Mediated Blood–Brain Barrier Opening Increases Cell-Free DNA in a Time-Dependent Manner. Neurooncol. Adv..

[31] Dong Q., He L., Chen L., Deng Q. (2018). Opening the Blood-Brain Barrier and Improving the Efficacy of Temozolomide Treatments of Glioblastoma Using Pulsed, Focused Ultrasound with a Microbubble Contrast Agent. Biomed. Res. Int..

[32] Zhu L., Nazeri A., Pacia C.P., Yue Y., Chen H. (2020). Focused Ultrasound for Safe and Effective Release of Brain Tumor Biomarkers into the Peripheral Circulation. PLoS ONE.

[33] Meng Y., Pople C.B., Suppiah S., Llinas M., Huang Y., Sahgal A., Perry J., Keith J., Davidson B., Hamani C. (2021). MR-Guided Focused Ultrasound Liquid Biopsy Enriches Circulating Biomarkers in Patients with Brain Tumors. Neuro Oncol..

[34] Pacia C.P., Yuan J., Yue Y., Xu L., Nazeri A., Desai R., Gach H.M., Wang X., Talcott M.R., Chaudhuri A.A. (2022). Sonobiopsy for Minimally Invasive, Spatiotemporally-Controlled, and Sensitive Detection of Glioblastoma-Derived Circulating Tumor DNA. Theranostics.

[35] Yuan J., Xu L., Chien C.-Y., Yang Y., Yue Y., Fadera S., Stark A.H., Schwetye K.E., Nazeri A., Desai R. (2023). First-in-Human Prospective Trial of Sonobiopsy in High-Grade Glioma Patients Using Neuronavigation-Guided Focused Ultrasound. NPJ Precis. Oncol..

[36] Wu S.K., Chu P.C., Chai W.Y., Kang S.T., Tsai C.H., Fan C.H., Yeh C.K., Liu H.L. (2017). Characterization of Different Microbubbles in Assisting Focused Ultrasound-Induced Blood-Brain Barrier Opening. Sci. Rep..

[37] McMahon D., Poon C., Hynynen K. (2019). Evaluating the Safety Profile of Focused Ultrasound and Microbubble-Mediated Treatments to Increase Blood-Brain Barrier Permeability. Expert Opin. Drug Deliv..

[38] Ahluwalia M., De Groot J., Lee J., Mogilner A., Schwartz T.H., Burns T., Shah B., McDermott M., Bettegowda C., Khosla A. (2022). Pivotal Study to Evaluate Safety and Efficacy of Exablate Model 4000 Using Microbubble Resonators to Temporarily Mediate Blood-Brain Barrier Disruption for Liquid Biopsy in Glioblastoma (LIBERATE). Neuro Oncol..

[39] Miller A., Ramamurthy B., Pike L., Keralapura M., Hofius J., Marshall J., Jones M., Lee C.-Y., Stember J., Brennan C. (2022). Focused Ultrasound Mediated Blood-Brain Barrier Penetrance to Enable Cell-Free DNA (CfDNA) as a Liquid Biopsy in Recurrent Primary Brain Tumors. Neuro Oncol..

[40] Sheybani N.D., Batts A.J., Mathew A.S., Andrew Thim E., Price R.J. (2020). Focused Ultrasound Hyperthermia Augments Release of Glioma-Derived Extracellular Vesicles with Differential Immunomodulatory Capacity. Theranostics.

[41] Newman A.M., Lovejoy A.F., Klass D.M., Kurtz D.M., Chabon J.J., Scherer F., Stehr H., Liu C.L., Bratman S.V., Say C. (2016). Integrated Digital Error Suppression for Improved Detection of Circulating Tumor DNA. Nat. Biotechnol..

[42] Zhang D.Y., Dmello C., Chen L., Arrieta V.A., Gonzalez-Buendia E., Robert Kane J., Magnusson L.P., Baran A., David James C., Horbinski C. (2020). Ultrasound-Mediated Delivery of Paclitaxel for Glioma: A Comparative Study of Distribution, Toxicity, and Efficacy of Albumin-Bound versus Cremophor Formulations. Clin. Cancer Res..

