# Supplementary material for: Focused Ultrasound-Enhanced Liquid Biopsy: A Promising Diagnostic Tool for Brain Tumor Patients
Source: Cancers (Basel). 2024 Apr 19;16(8):1576. doi: 10.3390/cancers16081576 (PMC11049441; doi:10.3390/cancers16081576)
Supplement: Supplementary file 1 [file cancers-16-01576-s001.zip › cancers-2946068-supplementary.docx]

**Table S1.** Search string in Pubmed and Embase.

| **Database** | **Search string** |
| --- | --- |
| PubMed | ((("Brain"[Mesh]) OR “Brain”[Title/Abstract]) OR (((((((((((((((“Glioblastoma*”[Title/Abstract]) OR GBM”[Title/Abstract]) OR “Glioma*”[Title/Abstract]) OR “Glial cell tumor*”[Title/Abstract]) OR “Glial cell tumour*”[Title/Abstract]) OR “Astrocytoma*”[Title/Abstract]) OR “Astroglioma*”[Title/Abstract]) OR “Oligodendroglioma*”[Title/Abstract]) OR “Oligoastrocytoma*”[Title/Abstract]) OR “LGG”[Title/Abstract]) OR “HGG”[Title/Abstract]) OR “Glioblastoma”[Mesh]) OR “Glioma”[Mesh]) OR “Astrocytoma”[Mesh]) OR “Oligodendroglioma”[Mesh]) OR ((“Polymorphous low-grade neuroepithelial tumor of the young”[Title/Abstract]) OR “PLNTY”[Title/Abstract]) OR (“Pleomorphic xanthoastrocytoma*”[Title/Abstract]) OR (((“Astroblastoma”[Title/Abstract]) OR “Astroblastomas”[Title/Abstract]) OR “Neoplasms, neuroepithelial”[Mesh]) OR (“Ganglioglioma*”[Title/Abstract]) OR ((((“Dysembryoplastic neuroepithelial tumor*”[Title/Abstract]) OR “Dysembryoplastic neuroepithelial tumour*”[Title/Abstract]) OR “DNET”[Title/Abstract]) OR “DNT”[Title/Abstract]) OR ((((((“Glioneuronal tumor*”[Title/Abstract]) OR “Glioneuronal tumour*”[Title/Abstract]) OR “Neuronal tumor*”[Title/Abstract]) OR “Neuronal tumour*”[Title/Abstract]) OR “Neuronal-glial tumor*”[Title/Abstract]) OR “Neuronal-glial tumour*”[Title/Abstract]) OR ((((“Gangliocytoma*”[Title/Abstract]) OR “DGC”[Title/Abstract]) OR “Lhermitte-Duclos disease”[Title/Abstract]) OR “LDD”[Title/Abstract]) OR (((“Multinodular and vacuolating neuronal tumor*”[Title/Abstract]) OR “Multinodular and vacuolating neuronal tumour*”[Title/Abstract]) OR “MVNT”[Title/Abstract]) OR ((((((“Ependymoma*”[Title/Abstract]) OR “Ependymal tumor*”[Title/Abstract]) OR “Ependymal tumour*”[Title/Abstract]) OR “Subependymoma”[Title/Abstract]) OR “Ependymal glioma*”[Title/Abstract]) OR "Ependymoma"[Mesh]) OR (((“Medulloblastoma*”[Title/Abstract]) OR “Medullo blastoma*”[Title/Abstract]) OR "Medulloblastoma"[Mesh]) OR (((((((“Pineal tumor*”[Title/Abstract]) OR “Pineal tumour*”[Title/Abstract]) OR “Pineocytoma*”[Title/Abstract]) OR “Pineoblastoma*”[Title/Abstract]) OR “Tumor* of pineal region”[Title/Abstract]) OR “Pinealoma”[Title/Abstract]) OR “Pinealoma"[Mesh]) OR ((((((“Hemangioblastoma*”[Title/Abstract]) OR “Hemangio-blastoma*”[Title/Abstract]) OR “Haemangioblastoma*”[Title/Abstract]) OR “Hemangioblastomatosis”[Title/Abstract]) OR “Haemangioblastomatosis”[Title/Abstract]) OR “Hemangioblastoma”[Mesh]) OR ((((“Central nervous system lymphoma*”[Title/Abstract]) OR “CNS lymphoma*”[Title/Abstract]) OR “Lymphoma* of central nervous system”[Title/Abstract]) OR “PCNSL”[Title/Abstract]) OR ((((((((((((((“Craniopharyngioma*”[Title/Abstract]) OR “Cranio pharyngioma*”[Title/Abstract]) OR “Craniopharyngeoma*”[Title/Abstract]) OR “Pituicytoma*”[Title/Abstract]) OR “Spindle cell oncocytoma*”[Title/Abstract]) OR “SCO”[Title/Abstract]) OR “Pituitary adenoma*”[Title/Abstract]) OR “Adenoma of the pituitary gland”[Title/Abstract]) OR “Hypophyseal adenoma*”[Title/Abstract]) OR “Pituitary gland adenoma*”[Title/Abstract]) OR “Hypophysis adenoma*”[Title/Abstract]) OR “Pituitary blastoma*”[Title/Abstract]) OR “Craniopharyngioma”[Mesh]) OR “Pituitary neoplasms”[Mesh]))  AND (((((((((((((((((((((((((((((“Biomarker*”[Text]) OR “Biological marker*”[Text]) OR “Biologic marker*”[Text]) OR “Biological marker*”[Text]) OR “Serum marker*”[Text] OR "Biomarkers"[Mesh]) OR “Cell free DNA”[Text]) OR “Cell-free DNA”[Text]) OR “cfDNA”[Text]) OR “cf-DNA”[Text]) OR “Tumor free DNA”[Text]) OR “Tumor-free DNA”[Text]) OR “Tumour free DNA”[Text]) OR “Tumour-free DNA”[Text]) OR “ctDNA”[Text]) OR “ct-DNA”[Text]) OR “Cell-free RNA”[Text]) OR “cfRNA”[Text]) OR “cf-RNA”[Text]) OR “Extracellular vesicles”[Text]) OR “EV”[Text]) OR “EVs”[Text]) OR “Circulating tumor cells”[Text]) OR “CTCs”[Text]) OR “CTC”[Text]) OR “Liquid biopsy*”[Text]) OR “LBx”[Text]) OR “Fluid biopsy*”[Text]) OR “Fluid phase biopsy*”[Text]) OR “Liquid biopsy”[Mesh])  AND (((((((((((((((((((((((((((((((((("Focused ultrasound"[Title/Abstract]) OR “FUS”[Title/Abstract]) OR “Magnetic resonance guided focused ultrasound”[Title/Abstract]) OR “Magnetic resonance imaging guided focused ultrasound”[Title/Abstract]) OR “MRI-guided focused ultrasound”[Title/Abstract]) OR “MRI-guided FUS”[Title/Abstract]) OR “MR-guided focused ultrasound”[Title/Abstract]) OR “MR-guided FUS”[Title/Abstract]) OR “MRgFUS”[Title/Abstract]) OR “MRIgFUS”[Title/Abstract]) OR “Focused ultrasound-enabled liquid biopsy”[Title/Abstract]) OR “FUS-LBx”[Title/Abstract]) OR “High-intensity focused ultrasound”[Title/Abstract]) OR “HIFU”[Title/Abstract]) OR “Magnetic resonance-guided high-intensity focused ultrasound”[Title/Abstract]) OR “Magnetic resonance imaging-guided high-intensity focused ultrasound”[Title/Abstract]) OR “Magnetic resonance guided high-intensity focused ultrasound”[Title/Abstract]) OR “Magnetic resonance imaging-guided high-intensity focused ultrasound”[Title/Abstract]) OR “MR-HIFU”[Title/Abstract]) OR “MRI-HIFU”[Title/Abstract]) OR “Low intensity focused ultrasound”[Title/Abstract]) OR “LOFU”[Title/Abstract]) OR “Microbubble-enhanced focused ultrasound”[Title/Abstract]) OR “Microbubble-assisted focused ultrasound”[Title/Abstract]) OR “MB-FUS”[Title/Abstract]) OR “Transcranial focused ultrasound”[Title/Abstract]) OR “tFUS”[Title/Abstract]) OR “Transcranial magnetic resonance-guided focused ultrasound”[Title/Abstract]) OR “Transcranial magnetic resonance imaging-guided focused ultrasound”[Title/Abstract]) OR “tcMRgFUS”[Title/Abstract] )OR “Sonobiopsy”[Title/Abstract])  OR “Sonoporation”[Title/Abstract])OR ("Ultrasound*"[Text] AND "Microbubbles"[Text]))OR “Ultrasound-mediated”[Title/Abstract]) |
| Embase | (((‘brain’) OR ‘brain’:ti,ab,kw) OR (((((((((((((((‘Glioblastoma*’:ti,ab,kw) OR ‘GBM’:ti,ab,kw) OR ‘Glioma*’:ti,ab,kw) OR ‘Glial cell tumor*’:ti,ab,kw) OR ‘Glial cell tumour*’:ti,ab,kw) OR ‘Astrocytoma*’:ti,ab,kw) OR ‘Astroglioma*’:ti,ab,kw) OR ‘Oligodendroglioma*’:ti,ab,kw) OR ‘Oligoastrocytoma*’:ti,ab,kw) OR ‘LGG’:ti,ab,kw) OR ‘HGG’:ti,ab,kw) OR ‘Glioblastoma’:ti,ab,kw) OR ‘Glioma’:ti,ab,kw) R ‘Astrocytoma’:ti,ab,kw) OR ‘Oligodendroglioma’:ti,ab,kw) OR (((‘Polymorphous low-grade neuroepithelial tumor of the young’:ti,ab,kw) OR ‘PLNTY’:ti,ab,kw) OR ‘Pleomorphic xanthoastrocytoma*’:ti,ab,kw) OR ((((‘Astroblastoma’:ti,ab,kw) OR ‘Astroblastomas’:ti,ab,kw) OR ‘Neuroepithelioma’:ti,ab,kw) OR ‘Ganglioglioma*’:ti,ab,kw) OR ((((‘Dysembryoplastic neuroepithelial tumor*’:ti,ab,kw) OR ‘Dysembryoplastic neuroepithelial tumour*’:ti,ab,kw) OR ‘DNET’:ti,ab,kw) OR ‘DNT’:ti,ab,kw) OR ((((((‘Glioneuronal tumor*’:ti,ab,kw) OR ‘Glioneuronal tumour*’:ti,ab,kw) OR ‘Neuronal tumor*’:ti,ab,kw) OR ‘Neuronal tumour*’:ti,ab,kw) OR ‘Neuronal-glial tumor*’:ti,ab,kw) OR ‘Neuronal-glial tumour*’:ti,ab,kw) OR ((((‘Gangliocytoma*’:ti,ab,kw) OR ‘DGC’:ti,ab,kw) OR ‘Lhermitte-Duclos disease’:ti,ab,kw) OR ‘LDD’:ti,ab,kw) OR (((‘Multinodular and vacuolating neuronal tumor*’:ti,ab,kw) OR ‘Multinodular and vacuolating neuronal tumour*’:ti,ab,kw) OR ‘MVNT’:ti,ab,kw) OR ((((((‘Ependymoma*’:ti,ab,kw) OR ‘Ependymal tumor*’:ti,ab,kw) OR ‘Ependymal tumour*’:ti,ab,kw) OR ‘Subependymoma’:ti,ab,kw) OR ‘Ependymal glioma*’:ti,ab,kw) OR ‘Ependymoma’) OR (((‘Medulloblastoma*’:ti,ab,kw) OR ‘Medullo blastoma*’:ti,ab,kw) OR ‘Medulloblastoma’) OR (((((((‘Pineal tumor*’:ti,ab,kw) OR ‘Pineal tumour*’:ti,ab,kw) OR ‘Pineocytoma*’:ti,ab,kw) OR ‘Pineoblastoma*’:ti,ab,kw) OR ‘Tumor* of pineal region’:ti,ab,kw) OR ‘Pinealoma’:ti,ab,kw) OR ‘Pineocytoma’) OR ((((((‘Hemangioblastoma*’:ti,ab,kw) OR ‘Hemangio-blastoma*’:ti,ab,kw) OR ‘Haemangioblastoma*’:ti,ab,kw) OR ‘Hemangioblastomatosis’:ti,ab,kw) OR ‘Haemangioblastomatosis’:ti,ab,kw) OR ‘Hemangioblastoma’) OR ((((‘Central nervous system lymphoma*’:ti,ab,kw) OR ‘CNS lymphoma*’:ti,ab,kw) OR ‘Lymphoma* of central nervous system’:ti,ab,kw) OR ‘PCNSL’:ti,ab,kw) OR ((((((((((((((‘Craniopharyngioma*’:ti,ab,kw) OR ‘Cranio pharyngioma*’:ti,ab,kw) OR ‘Craniopharyngeoma*’:ti,ab,kw) OR ‘Pituicytoma*’:ti,ab,kw) OR ‘Spindle cell oncocytoma*’:ti,ab,kw) OR ‘SCO’:ti,ab,kw) OR ‘Pituitary adenoma*’:ti,ab,kw) OR ‘Adenoma of the pituitary gland’:ti,ab,kw) OR ‘Hypophyseal adenoma*’:ti,ab,kw) OR ‘Pituitary gland adenoma*’:ti,ab,kw) OR ‘Hypophysis adenoma*’:ti,ab,kw) OR ‘Pituitary blastoma*’:ti,ab,kw) OR ‘Craniopharyngioma’) OR ‘Pituitary neoplasm’) )  AND ((((((((((((((((((((((((((((‘Biomarker*’:ti,ab,kw) OR ‘Biological marker*’:ti,ab,kw) OR ‘Biologic marker*’:ti,ab,kw) OR ‘Serum marker*’:ti,ab,kw) OR ‘Biological marker’:ti,ab,kw) OR ‘Cell-free DNA’:ti,ab,kw) OR ‘cfDNA’:ti,ab,kw) OR ‘cf-DNA’:ti,ab,kw) OR ‘Tumour-free DNA’:ti,ab,kw) OR ‘Tumor-free DNA’:ti,ab,kw) OR ‘Tumour free DNA’:ti,ab,kw) OR ‘Tumor free DNA’:ti,ab,kw) OR ‘ctDNA’:ti,ab,kw) OR ‘ct-DNA’:ti,ab,kw) OR ‘Cell-free RNA’:ti,ab,kw) OR ‘cfRNA’:ti,ab,kw) OR ‘cf-RNA’:ti,ab,kw) OR ‘Extracellular vesicles’:ti,ab,kw) OR ‘EV’:ti,ab,kw) OR ‘EVs’:ti,ab,kw) OR ‘Circulating tumor cells’:ti,ab,kw) OR ‘CTCs’:ti,ab,kw) OR ‘CTC’:ti,ab,kw) OR ‘Liquid biopsy*’:ti,ab,kw) OR ‘LBx’:ti,ab,kw) OR ‘Fluid biopsy*’:ti,ab,kw) OR ‘Fluid phase biopsy*’:ti,ab,kw) OR ‘Liquid biopsy’:ti,ab,kw)  AND ((((((((((((((((((((((((((((((((((((‘Focused ultrasound’:ti,ab,kw) OR ‘FUS’:ti,ab,kw) OR ‘Magnetic resonance guided focused ultrasound’:ti,ab,kw) OR ‘Magnetic resonance imaging guided focused ultrasound’:ti,ab,kw) OR ‘MRI-guided focused ultrasound’:ti,ab,kw) OR ‘MRI-guided FUS’:ti,ab,kw) OR ‘MR-guided focused ultrasound’:ti,ab,kw) OR ‘MR-guided FUS’:ti,ab,kw) OR ‘MRgFUS’:ti,ab,kw) OR ‘MRIgFUS’:ti,ab,kw) OR ‘Focused ultrasound-enabled liquid biopsy’:ti,ab,kw) OR ‘FUS-LBx’:ti,ab,kw) OR ‘High-intensity focused ultrasound’:ti,ab,kw) OR ‘HIFU’:ti,ab,kw) OR ‘Magnetic resonance-guided high-intensity focused ultrasound’:ti,ab,kw) OR ‘Magnetic resonance imaging-guided high-intensity focused ultrasound’:ti,ab,kw) OR ‘Magnetic resonance guided high-intensity focused ultrasound’:ti,ab,kw) OR ‘Magnetic resonance imaging-guided high-intensity focused ultrasound’:ti,ab,kw) OR ‘MR-HIFU’:ti,ab,kw) OR ‘MRI-HIFU’:ti,ab,kw) OR ‘Low intensity focused ultrasound’:ti,ab,kw) OR ‘LOFU’:ti,ab,kw) OR ‘Microbubble-enhanced focused ultrasound’:ti,ab,kw) OR ‘Microbubble-assisted focused ultrasound’:ti,ab,kw) OR ‘MB-FUS’:ti,ab,kw) OR ‘Transcranial focused ultrasound’:ti,ab,kw) OR ‘tFUS’:ti,ab,kw) OR ‘Transcranial magnetic resonance-guided focused ultrasound’:ti,ab,kw) OR ‘Transcranial magnetic resonance imaging-guided focused ultrasound’:ti,ab,kw) OR ‘tcMRgFUS’:ti,ab,kw) OR ‘MR-guided focused ultrasound’:ti,ab,kw) OR ‘Transcranial magnetic resonance guided focused ultrasound’:ti,ab,kw) OR ‘Sonobiopsy’:ti,ab,kw) OR ‘Sonoporation’:ti,ab,kw) OR (‘Ultrasound*’:ti,ab,kw AND ‘Microbubbles’:ti,ab,kw)) OR ‘Ultrasound-mediated’:ti,ab,kw) |

**Table S2.** Characteristics and results of the preclinical studies included in the systematic review [24,30–32,34]. Data were also retrieved from [42]. *) Mice numbers of [30] were estimated from the figures in the article. **) likely no hemorrhaging was found because of the short time interval between sonication and sacrifice of the model. Abbreviations: FUS= Focused ultrasound; FWHM= Full width half maximum; MR= magnetic resonance; US= ultrasound; ddPCR= digital droplet polymerase chain reaction; qPCR= quantitative polymerase chain reaction.

|  | **Zhang et al. [30]** | | **Dong et al. [31]** | **Pacia et al. [34]** | | **Zhu et al. [24]** | | **Zhu et al. [32]** |
| --- | --- | --- | --- | --- | --- | --- | --- | --- |
|  |  |  |  |  |  |  |  |  |
| Animal | Healthy mice | C57/ BL6 mice | Wistar rats | Immunodeficient mice | Pigs | NCl athymic NCr-nu/nu mice | NIH Swiss mice | NIH Swiss mice |
| Strain | n.r. | n.r. | n.r. | CU Athymic NCR-nu/nu | Yorkshire white | Strain 553 | Strain 550 | Strain 550 |
| Supplier | n.r. | n.r. | Huafukang Biotechnology Company (Beijing, China) | Charles River Laboratory (Wilmington, MA, USA) | Oak Hill Genetics (Ewing, IL, USA) | Charles River Laboratory (Wilmington, MA, USA) | | Charles River Laboratory (Wilmington, MA, USA) |
| Total amount of animals | ≥95 * | ≥62 * | 20 | 45 | 10 | 9 | 12 | 20 |
| Animals that received FUS | ≥71 * | ≥46 * | 10 | 24 | 10 | 6 | 9 | 15 |
| Control animals | ≥24 * | ≥16 * | 10 | 21 | NA | 3 | 3 | 5 |
| Age | n.r. | n.r. | n.r. | 6-8 weeks | 4 weeks | n.r. | n.r. | 6-8 weeks |
| Weight | n.r. | n.r. | 200 ± 20 gr | n.r. | 15 lbs | n.r. | n.r. | n.r. |
| Sex | n.r. | n.r. | n.r. | n.r. | male | n.r. | n.r. | female |
| Tumor cell line | NA | PF8 murine glioma | 9L glioma | U87 human glioblastoma | U87 human glioblastoma | U87 human glioblastoma | GL261 murine glioblastoma | GL261 murine glioblastoma |
| Amount of cells | NA | 10000 | 10^6 | n.r. | 3 x 10^6 | n.r. | n.r. | >50000 |
| Location tumor | NA | intracranial | brain striatum | intracranial | bilateral in cortex | intracranial | intracranial | intracranial |
|  |  |  |  |  |  |  |  |  |
| Drug | NA | | Temozolomide (TMZ) | NA | NA | NA | NA | NA |
| Dose | NA | | 5 days 100 mg/kg | NA | NA | NA | NA | NA |
|  |  |  |  |  |  |  |  |  |
| Elements | n.r. | | 80 | 7 | 15 | n.r. | 256 | 256 (128 used) |
| Transducer geometry | n.r. | | Phased array | Annular array | n.r. | n.r. | Phased array | Phased array |
| Frequency (MHz) | 1 | | 1.7/3.3 | 1.5 | 0.65 | 1.5 | 1.44 | 1.44 |
| FWHM axial (mm) | n.r. | | n.r. | 5.5 | 3 | 6.04 | 12.1 | 12.1 |
| FWHM lateral (mm) | n.r. | | n.r. | 1.2 | 20 | 0.62 | 1.37 | 1.37 |
| System | Preclinical SonoCloud | | IE33 with S5-1 probe |  |  | VIFU 2000 | Sonalleve V2 | Sonalleve V2 |
| Treatment guided by | n.r. | | n.r. | MR | MR | US | MR | MR |
| Manufacturer | Carthera (France) | | Philips Healthcare (Best, the Netherlands) | Imasonics (Voray sur l'Ognon, France) | Image Guided Therapy (Pessac France) | Alpinion US Inc. (Bothell, WA, USA) | Profound Medical Inc. (Mississauga, Canada) | Profound Medical Inc. (Mississauga, Canada) |
|  |  |  |  |  |  |  |  |  |
| Acoustic pressure (MPa) | 0.3 and 0.4 | 0.4 | n.r. | 1 | 3 | 3.82 | 1.52, 2.74 and 3.53 | 0.59, 1.29 and 1.58 |
| Pulse length (ms) | 25 | | n.r. | 6.7 | 10 | 10 | 10 | 10 |
| Pulse repetition frequency (Hz) | 1 | | n.r. | 5 | 1 | 1 | 1 | 1 |
| Treatment duration (min) | 2 | | 10 | 3 | 3 | 2 | 2 | 4 |
| Treatments | 1 | 1 and 2 | 5 x daily (after TMZ) | 1 | 2 (1 hr apart) | 1 | 1 | 1 |
| Mechanical index | 0.3 and 0.4 | 0.4 | 0.8 | 0.8 | 2.5 | 3.1 | 1.3, 2.3 and 2.9 | 0.5, 1.1 and 1.3 |
|  |  |  |  |  |  |  |  |  |
| Name | Lumason | | n.r. | Definity | | NA | | NA |
| Manufacturer | Bracco | | Third Military Medical University | Lantheus Medical Imaging | | In-house | | In-house |
| Gas and shell | Sulfur hexafluoride gas + lipid shell | | Sulfur hexafluoride gas | Perflutren gas + lipid shell | | Perfluorobutane gas + lipid shell | | Perfluorobutane gas + lipid shell |
| Dose | 10 mg/kg | | 1 mg/kg | 100 µl/kg | 20 µl/kg | 30 µl (24 x 10^6 MBs) | | 30 µl (24 x 10^6 MBs) |
|  |  |  |  |  |  |  |  |  |
| Volume (mL) | 0.5 - 1.0 | | 0.5 | 0.5 | 10 | 0.5 - 0.8 | 0.5 - 0.8 | 0.5 - 0.8 |
| Location | Heart | | Angular vein | Heart | Peripheral vessel | Heart | Heart | Heart |
| Timing | 2, 15, 30, 45, 60 min and 24 h | 2, 10, and 45 min | direct | 10 min | 10 min | 4 min | 20 min | 20 min |
| Detection methods | ddPCR, Qubit high sensitivity dsDNA assay | | ELISA | ddPCR, Qubit Fluorometer | | qPCR | qPCR | qPCR |
|  |  |  |  |  |  |  |  |  |
| Biomarker 1 | cfDNA (0 -280 bp) | cfDNA (0 -280 bp) | Protein: GFAP | EGFRvIII ctDNA | EGFRvIII ctDNA | eGFP primer A mRNA | eGFP primer A mRNA | eGFP primer A mRNA |
| X-fold change (post-/pre-FUS) | 1.1 - 3.9 | 1.1 - 8.1 | 1.6 | 920 | 270 | 17560 | 2702 - 4096 | 55 - 8372 |
| Biomarker 2 |  |  |  | TERT C228T | TERT C228T | eGFP primer B mRNA | eGFP primer B mRNA | eGFP primer B mRNA |
| X-fold increase (post-/pre-FUS) |  |  |  | 10 | 9 | 2521 | 955 - 2702 | 221 - 4793 |
| Hemorrhaging | n.r. | | n.r. | Not significant | Not significant | No ** | Major - severe | No, minor and major |

**Table S3.** Risk of bias of the preclinical studies assessed with SYRCLE’s (SYstematic Review Centre for Laboratory animal Experimentation) RoB (Risk of Bias) tool (released in 2014). Website: https://www.syrcle.network/

| Major Components | **Dong et al. [31]** | Pacia et al. [34] | **Zhang et al. [30]** | **Zhu et al. [24]** | **Zhu et al. [32]** |
| --- | --- | --- | --- | --- | --- |
| 1. Was the allocation sequence adequately generated and applied?  Did the investigators describe a random component in the sequence generation process such as: Referring to a random number table; Using a computer random number generator | Unclear | Unclear | Unclear | Unclear | No |
| 2. Were the groups similar at baseline or were they adjusted for confounders in the analysis?   - Was the distribution of relevant baseline characteristics balanced for the intervention and control groups? - If relevant, did the investigators adequately adjust for unequal distribution of some relevant baseline characteristics in the analysis? - Was the timing of disease induction adequate? | Unclear | Yes | Yes | Yes | Unclear |
| 3. Was the allocation to the different groups adequately concealed during?   - Could the investigator allocating the animals to intervention or control group not foresee assignment due to one of the following or equivalent methods? Third-party coding of experimental and control group allocation Central randomization by a third party Sequentially numbered opaque, sealed envelopes | Unclear | Unclear | Unclear | Unclear | Unclear |
| 4. Were the animals randomly housed during the experiment?   - Did the authors randomly place the cages or animals within the animal room/facility? Animals were selected at random during outcome assessment (use signaling questions of entry 6). - Is it unlikely that the outcome or the outcome measurement was influenced by not randomly housing the animals? | Unclear | Unclear | Unclear | Unclear | Yes |
| 5. Were the caregivers and/or investigators blinded from knowledge which intervention each animal received during the experiment?   - Was blinding of caregivers and investigators ensured, and was it unlikely that their blinding could have been broken? ID cards of individual animals, or cage/animal labels are coded and identical in appearance; Sequentially numbered drug containers are identical in appearance; The circumstances during the intervention are specified and similar in both groups (#).; Housing conditions of the animals during the experiment are randomized within the room (use criteria of entry 4). | Unclear | Unclear | Unclear | Unclear | Unclear |
| The relevance of the above-mentioned items depends on the experiment. Authors of the review need to judge for themselves which of the above-mentioned items could cause bias in the results when not similar. These should be assessed. | | | | | |
| 6. Were animals selected at random for outcome assessment?   - Did the investigators randomly pick an animal during outcome assessment, or did they use a random component in the sequence generation for outcome assessment? Referring to a random number table; Using a computer random number generator; Etc. | Unclear | Unclear | Yes | Unclear | Yes |
| 7. Was the outcome assessor blinded?   - Was blinding of the outcome assessor ensured, and was it unlikely that blinding could have been broken? Outcome assessment methods were the same in both groups; Animals were selected at random during outcome assessment (use signaling questions of entry 6). - Was the outcome assessor not blinded, but do review authors judge that the outcome is not likely to be influenced by lack of blinding? (e.g., mortality) | Unclear | Unclear | Unclear | Yes | Unclear |
| 8. Were incomplete outcome data adequately addressed?   - Were all animals included in the analysis? - Were the reasons for missing outcome data unlikely to be related to true outcome? (e.g., technical failure) - Are missing outcome data balanced in numbers across intervention groups, with similar reasons for missing data across groups? - Are missing outcome data imputed using appropriate methods? | Yes | Unclear | Unclear | Yes | Yes |
| 9. Are reports of the study free of selective outcome reporting?   - Was the study protocol available and were all of the study’s pre-specified primary and secondary outcomes reported in the current manuscript? - Was the study protocol not available, but was it clear that the published report included all expected outcomes (i.e. comparing methods and results section)? | No | Yes | No | Yes | Yes |
| 10. Was the study apparently free of other problems that could result in high risk of bias?   - Was the study free of contamination (pooling drugs)? - Was the study free of inappropriate influence of funders? - Was the study free of unit of analysis errors? - Were design-specific risks of bias absent? - Were new animals added to the control and experimental groups to replace drop-outs from the original population? | Yes | Yes | Yes | Yes | Yes |
| **Risk of bias assessment** | **High** | **High** | **High** | **High** | **High** |

**Table S4.** Risk of bias of the clinical studies assessed with the National Institutes of Health (NIH) quality assessment tool for before-after (Pre-Post) study with no control group. Website: https://www.nhlbi.nih.gov/health-topics/study-quality-assessment-tools.

|  | | |
| --- | --- | --- |
| Major Components | Meng et al. [33] | **Yuan et al. [35]** |
| 1. Was the study question or objective clearly stated? | Yes | Yes |
| 2. Were eligibility/selection criteria for the study population prespecified and clearly described? | Yes | Yes |
| 3. Were the participants in the study representative of those who would be eligible for the test/service/intervention in the general or clinical population of interest? | Yes | Yes |
| 4. Were all eligible participants that met the prespecified entry criteria enrolled? | No | No |
| 5. Was the sample size sufficiently large to provide confidence in the findings? | Yes | Yes |
| 6. Was the test/service/intervention clearly described and delivered consistently across the study population? | Yes | Yes |
| 7. Were the outcome measures prespecified, clearly defined, valid, reliable, and assessed consistently across all study participants? | Yes | Yes |
| 8. Were the people assessing the outcomes blinded to the participants' exposures/interventions? | Not Reported | Not Reported |
| 9. Was the loss to follow-up after baseline 20% or less? Were those lost to follow-up accounted for in the analysis? | Not Reported | Not Applicable |
| 10. Did the statistical methods examine changes in outcome measures from before to after the intervention? Were statistical tests done that provided p values for the pre-to-post changes? | Yes | Yes |
| 11. Were outcome measures of interest taken multiple times before the intervention and multiple times after the intervention (i.e., did they use an interrupted time-series design)? | No | No, only post-FUS multiple times |
| 12. If the intervention was conducted at a group level (e.g., a whole hospital, a community, etc.) did the statistical analysis take into account the use of individual-level data to determine effects at the group level? | Not Applicable | Not Applicable |
| **Quality Rating** | Fair | Fair |
